# Overcoming the Limits of Ejection Fraction and Ventricular-Arterial Coupling in Heart Failure

**DOI:** 10.3389/fcvm.2021.750965

**Published:** 2022-01-21

**Authors:** Elena-Laura Antohi, Ovidiu Chioncel, Serban Mihaileanu

**Affiliations:** ^1^Emergency Institute for Cardiovascular Diseases “Prof. Dr. C.C. Iliescu”, Bucharest, Romania; ^2^“Carol Davila” University of Medicine and Pharmacy, Bucharest, Romania; ^3^Institut Mutualiste Montsouris, Paris, France

**Keywords:** heart failure, ventricular-arterial coupling, left ventricular ejection fraction, systolic times, left ventricular elastance, blood pressure

## Abstract

Left ventricular ejection fraction (LVEF) and ventricular-arterial coupling (VAC) [VAC = Ea/Ees; Ea: effective arterial elastance; Ees: left ventricle (LV) elastance] are both dimensionless ratios with important limitations, especially in heart failure setting. The LVEF to VAC relationship is a divergent non-linear function, having a point of intersection at the specific value of 0.62, where V0 = 0 ml (V0: the theoretical extrapolated value of the volume-axis intercept at end-systolic pressure 0 mmHg). For the dilated LV, both LVEF and VAC are highly dependent on V0 which is inconclusive when derived from single-beat Ees formulas. VAC simplification should be avoided. Revisiting the relationship between systolic time intervals (STI), pressure, and volumes could provide simple-to-use guiding formulas, affordable for daily clinical practice. We have analyzed by echocardiography the hemodynamics of 21 patients with severe symptomatic heart failure with reduced ejection (HFrEF) compared to 12 asymptomatic patients (at risk of heart failure with mild structural disease). The groups were unequivocally separated by ‘classic’ measures (LVEF, LV end-systolic volume (ESV), LV mass, STI). Chen's Ees formula was weakly correlated with LVEF and indexed ESV (ESVi) but better correlated to the pre-ejection period (PEP); PEP/total ejection time (PEP/TET); systolic blood pressure/PEP (SBP/PEP) (*P* < 0.001). Combining the predictability of the LVEF to the determinant role of SBP/PEP on the Ees variations, we obtained: (SBP^*^LVEF)/PEP mm Hg/ms, with an improved *R*^2^ value (*R*^2^ = 0.848; *P* < 0.001). The strongest correlations to VAC were for LVEF (*R* = −0.849; *R*^2^ = 0.722) and PEP/TET (*R* = 0.925; *R*^2^ = 0.857). By multiple regression, the VAC was strongly predicted (*N* = 33): (*R* = 0.975; *R*^2^ = 0.95): VAC = 0.553–0.009^*^LVEF + 3.463^*^PEP/TET, and natural logarithm: Ln (VAC) = 0.147–1.4563^*^DBP/SBP^*^0.9–0.010^*^LVEF + 4.207^*^PEP/TET (*R* = 0.987; *R*^2^ = 0.975; *P* = 0) demonstrating its exclusive determinants: LVEF, PEP/TET, and DBP/SBP. Considering Ea as a known value, the VAC-derived Ees formula: Ees_d ≈ Ea/(0.553–0.009^*^LVEF+3.463^*^PEP/TET) was strongly correlated to Chen's Ees formula (*R* = 0.973; *R*^2^ = 0.947) being based on SBP, ESV, LVEF, and PEP/TET and no exponential power. Thus, the new index supports our hypothesis, in the limited sample of patients with HFrEF. Indices like SBP/PEP, (SBP^*^LVEF)/PEP, PEP/TET, and DBP/SBP deserve further experiments, underlining the major role of the forgotten STI.

## Introduction

Heart failure (HF) represents a complex clinical syndrome with a heterogeneous clinical and hemodynamic presentation. It encompasses a variety of etiologies, and its spectrum of severity is a continuum from “at risk” to patients with the most severe “advanced” HF, as recognized by the current stadialization of HF (1). Moreover, nowadays, most patients with HF are already being administered some ambulatory treatment with neurohormonal medications that potentially impact hemodynamics [i.e., scarce preliminary evidence suggests sacubitril/valsartan could improve ventricular-arterial coupling (2), pending more consistent results (ARNI-PVA trial, ClinicalTrials.gov Identifier NCT04498780)]. The heart is just part of a complex intricated system, the peripheral mechanism/response (including preload and afterload) being determinant. Therefore, hemodynamic characterization remains a challenge.

The current European Society of Cardiology HF guidelines supports the use of clinical hemodynamic profiling when assessing patients with acute HF (3) but for the patients with severe, advanced HF (stage D), it was shown that clinical examination only cannot suffice (4). More accurate hemodynamic characterization is important.

## Current Hemodynamic Characterization in Clinical Practice—Uses and Limitations

### Dimensionless Ratios

Left ventricular ejection fraction (LVEF) represents the centerpiece parameter used for the formal classification of HF and the identification of best candidates for the response to the available neurohormonal therapies (1, 3). It has the advantage of ease for being used in clinical practice and offers essential information on global cardiac performance and prognosis.

But LVEF has serious disadvantages as well, including methodological, technical, and clinical issues (5). Also, from a clinical standpoint, the HF classification into preserved, moderately reduced and reduced LVEF is limited in predicting outcome. An often-cited study by Shah et al. showed that 5-year mortality for hospitalized patients with HF does not differ significantly among the 3 groups (6). Among the most severe patients, LVEF is outperformed by other echocardiographic more physiologically robust indices (7). Kerkhof et al. previously described the limitations of LVEF as a dimensionless index ratio when compared to the more informative indexed left ventricular end-systolic volume (ESVi) for the assessment of LV function (8, 9).

Ventricular-arterial coupling (VAC) is another dimensionless index that gained relevance in chronic patients with HF as it was correlated with outcomes (10, 11); in an acute setting, vasoactive medications were tested to improve it (12–16). As it represents the ratio between effective arterial elastance (Ea) and ventricular elastance (Ees), it is regarded as being indicative of global cardiovascular performance. While VAC has a dynamic response in HF with reduced ejection fraction (EF) (elevated values with the decreased Ees and increased Ea), its meaning as a nude dimensionless number is not as informative in HF with preserved EF (in which case both Ees and Ea are elevated giving a ‘normal’ VAC) (17–19). Several issues have limited the widespread clinical use of VAC.

Invasive cardiology has become today, in common practice, quite exclusively therapeutic. Chen, Shishido, and Senzaki (20, 21) imagined Ees surrogate single-beat steady-state formulas, based on ultrasound examination. Non-invasive calculation of Ees relies on complex formulas, for which minimal measurement errors may be exponentially replicated (22). However, Chen's formula, the most popular, was validated by the original study and others (23). All the different single-beat formulas are based on systolic-time intervals, blood pressure, and LV volumes. But the linearity of this function has been questioned and it was suggested that with large load manipulation, Ees will vary non-linearly.

Left ventricular ejection fraction can be expressed as a relationship between left Ees, Ea, end-systolic pressure (ESP), and V0 (LVEF = $\frac{ESP^{*}Ees}{ESP\left(Ea+Ees\right)+Ea^{*}Ees^{*}V0}$), where V0 is an extrapolated value, representing the unstressed LV volume intercept of the volume-axis at a theoretical end-systolic pressure of 0 mm Hg. It was also mathematically demonstrated that V0 has a 0 ml value, only when LVEF (expressed in decimals) and VAC are equal at 0.62 (approximating φ = 0.618…) (23). Vo is significantly increased in dilated and dysfunctional ESV, being associated with prognosis in HF (11). However, its significance is controversial, as Vo is only a virtual theoretical value, extrapolated from research invasive studies. Meanwhile, available Vo formulas derived from single-beat determinations non-invasive data have wide, uncertain margins (24).

In order to calculate the VAC, another surrogate is needed: the components of the total arterial load were grouped in an all-in-one simple ratio (ESP/SV) (SV: Stroke Volume) named Ea (25). Ventricular-arterial coupling (VAC = Ea/Ees) is highly dependent on the V0 value as well. In the normal non-dilated LV with an LVEF close to 60%, V0 may be approximated at 0 ml, but by this way, the VAC will be reduced to a simple volumetric ratio (if V0 = 0, then VAC = $\frac{ESV\left(ml\right)}{SV\left(ml\right)}$), thus, failing to integrate significant hemodynamic determinants such as pressure. However, VAC has a more dynamic behavior than LVEF being a valuable tool in heart failure with reduced ejection fraction (HFrEF) and/or unstable hemodynamic situations. LVEF and VAC have a well-proven negative correlation but are not reducible to a simple formula (24). Less frequently, the VAC is calculated by the inverse ratio (Ees/Ea), the relation between the two ratios being like comparing *x* to *1/x*.

Systolic time intervals (STI) have long been proven as highly valuable for LV function study. PEP/LVET or PEP/TET ratio (pre-ejection period over left ventricular ejection time or total ejection time) is another dimensionless ratio with the ability to identify severe HF patients; the ratio itself and systolic ejection time have been proven to correlate with reduced EF and severe outcome (26, 27).

### Dimensionless Ratios: A Step Away, but Not Too Far…

Generally speaking, dimensionless ratios have certain disadvantages but bear a hidden practical quality: being dimensionless they can be added to other formulas without changing the units.

Several indices have been proposed to improve the sensitivity of LVEF, VAC, or its components (i.e., adjusting LVEF to afterload, adjusting Ees to Vo, dynamic arterial elastance), but without documented clinical value until so far (28, 29). On the basis of a robust analytical expression, it has been documented that EF is almost exclusively determined by ESV (30). Thus, the inclusion of a pressure-related term (such as ESP) while considering either EF or ESV is likely to contribute to a more comprehensive description of cardiac (patho)physiology. In fact, the popular metric Ees is precisely defined as ESP divided by the key variable ESV and additionally carries clear physical dimensions reflecting pump characteristics as typically seen in pressure-volume-loops (31).

The analysis of LV pressure-volume loops (PVL) points to the main parameters that rule cardiovascular hemodynamics: volumes, pressure, and time intervals. It is not surprising that data is gathered regarding the improved clinical utility of cardiac power output (CPO) or stroke work index (SWI).

Cardiac power output (CPO) represents the interplay between pressure, flow and heart rate and is, therefore, a good metric of the physics of cardiac pump efficiency—the latter being another dimensionless ratio with equivocal meaning difficult to interpret. In an acute setting, the CPO simplified formula (CPO = MAP × CO/451) as measured by pulmonary artery catheter, is sensitive enough to interventions and was found to be the most accurate predictor of death in the landmark SHOCK trial hemodynamic analysis (32). Invasive CPO, indexed CPO (CPI) measurements are predictive of outcomes in advanced chronic patients with HF as well (33, 34). The non-invasive echocardiographic calculation is equally feasible and both the simplified and the adjusted formula to mass have prognostic meaning; CPO reserve by stress testing (whether exercise or dobutamine) has been reported to predict prognosis better than LVEF with good reproducibility (35–38). Animal models and in-human research showed a good correlation for CPO with PVL-derived LV stroke work (LVSW) for various inotropic states. Sophisticated echocardiographic softwares allow for non-invasive global and regional myocardial stroke work calculation derived from pressure-strain loop analysis (39). But simpler estimation for indexed LVSW (LVSWi) is feasible and clinically relevant. Jentzer et al. recently showed on an impressive number of 4,536 patients admitted to a cardiac intensive care unit, that low LVSWI (calculated by echocardiography), predicts increased mortality risk and outperforms LVEF (7).

Ventricular elastance (Ees) is a very strong concept, inescapable for the VAC calculation which is, like for any engine, the great ruler of the dynamic equilibrium between the LV contraction stiffness, against the peripheral impedance—being a governing principle in HFrEF and/or any hemodynamic unstable situation. Ees is a non-linear time-varying function. Approximation of the Ees by single-beat surrogates is just a glimpse of its behavior—but much better than nothing. However single-beat formulas are underused in clinical practice, probably due to their complexity. Chen's Ees formula, the most popular, is highly sensitive to STI:

***Ees* = *(DBP* –* (End(est)* × *SBP* × *0.9))/End(est)* × *SV***

***End(est****)* = *0.0275 – 0.165* × *LVEF* + *0.3656* × *(DBP/SBP* × *0.9)* + *0.515* × *End(avg)*.

***End(avg)*** = *0.35695 – 7.2266* × *tNd* + *74.249* × *tNd*^2^ −*307.39* × *tNd* ^3^ + *684.54* × *tNd* ^4^ –* 856.92* × *tNd*^5^+ *571.95* × *tNd* ^6^ –* 159.1* × *tNd* ^7^

(tNd is the ratio of pre-ejection time to total systolic time). “tNd” is used from 1 to its 7th order! A 5 ms variation for the isovolumic contraction time can lead up to a 9% variation of the calculated Ees (22).

### Hypothesis

1. The VAC and LVEF relationship:
  a. The VAC and LVEF (calculated in decimals) have a divergent nonlinear relationship with an intersection point at 0.62 when V0 = 0.
  b. The formula relating the VAC to LVEF: VAC = Ea/Ees = 1/LVEF – 1, does not correctly predict the VAC if V0 deviates from zero.
  c. The simplified VAC formula, considering V0 = 0 ml (ESV/SV): does not correctly predict the VAC.
  d. The simplified arterial elastance formula, defined as effective arterial elastance
    Ea = (SBP^*^0.9)/SV, is strongly criticized, neglecting important determinants such as the reflected wave, heart frequency, and vascular stiffness. We investigated, for our group of patients, if Ea could be a good surrogate, comparing it with a more complex arterial elastance formula.
  e. The VAC value is obtained once Ea and Ees have been already calculated. However, more simple determinants could approach the VAC value.
2. Returning to the conceptual roots of Ees single-beat formula, we consider that the relationship between systolic times, pressure, and volumes needs to be revisited to provide simple-to-use guiding Ees approximations, approachable in daily clinical practice.

Echocardiographic indices that are readily available by simple and robust bedside measurements, independent of the quality of the acoustic window, having a physiological meaning, possibly incorporating the relevant systolic parameters mentioned before, remain necessary. HF hemodynamic characterization is counted among the current gaps in knowledge in HF documents (1). Adding pressure and time measurements to the LVEF (as a dimensionless ratio) could build reinforced hemodynamic indices.

### Supportive Clinical Data

#### Objective

a. We investigated the relationships: LVEF to VAC and VAC to its companion (VACC) = √(Ea^2^ + Ees^2^).
b. We have tested different indices of cardiovascular hemodynamics calculated by transthoracic echocardiography, to assess their ability to predict symptomatic HF, beyond LVEF. These included Ees, Ea, VAC, CPO, and STI.
c. We compared, in our group of patients, the simplified Ea formula to the more complex Segers, assuming a three-element windkessel model (40).
d. We searched for possible strong correlations between simple measurements and the VAC (calculated on the basis of Ea and Chen's Ees formula).
e. We tested the relationship between Chen's Ees formula and simple formulas based on systolic time intervals, pressure, and volumes, aiming for a more affordable estimation of the results obtained by this formula.

#### Methods

We investigated 33 patients, with valid echocardiographic evaluations (which were performed for routine clinical indications), divided into two groups by clinical characteristics, using the current HF clinical staging (1):

—group A – 12 patients at risk for HF or with asymptomatic HF (stages A and B); patients were either hypertensive or had only mild diastolic or systolic LV dysfunction and no clinical symptoms (in patients with cardiac structural abnormalities, normal cardiac biomarkers were documented);
—group B – 21 patients with symptomatic HF (stage C) and advanced HF with reduced EF (stage D); all patients in this group were New York Heart Association classes III and IV, having had a recent (in the past month) significant HF hospitalization; they were analyzed when free of systemic congestion, but significantly elevated natriuretic peptides were still documented (mean value for NT-proBNP 3326 +/– 192 pg/ml).

Patients with significant left-sided valvular disease, atrial fibrillation, and/or wide QRS (>130 ms) were excluded.

Standard echocardiography, including the following measurements and derived calculations, was performed in all patients:

—LVEF was calculated by biplane Simpson formula;
—pulsed-wave Doppler in the LV outflow tract (LVOT) was recorded at the same time with the right arm cuff blood pressure measurement; time-velocity integral (VTI) in the LVOT was calculated and stroke volume (SV) was derived;
—systolic times: pre-ejection period (PEP), LV ejection time (LVET), PEP/LVET, total ejection time (TET = PEP + LVET)), time from the beginning of QRS to peak velocity in the outflow tract (T+), time from peak velocity (Vmax) to the end of ejection (T-);
—The CPO was calculated non-invasively by the formula CPO = MAP x CO/451 (MAP is mean arterial pressure and CO is cardiac output) (32, 34, 37, 41);
—Segers formula $\text{Ea}=-0.127+1.02R/T+0.314/C=-0.127+1.023^{*}\frac{(MAP-CVP)/CO}{T(cyclelength)}+(0.314/\;\frac{SV}{PP})$, according to Chemla et al. (42); CVP was approximated by the inferior vena cava collapsibility index.
—Ees was calculated by Chen's formula; Ea by the SBP/SV ratio; VAC by Ea/Ees (21, 25, 43);
—Newly investigated ratios: SBP/PEP, PP/PEP, (SBP^*^LVEF)/PEP, VAC/LVEF (SBP: Systolic Blood Pressure; PP: Pulse Pressure, φ = 0.618… ≈0.62). With the exception of VAC/LVEF, all of them are expressed in mm Hg/ms. SBP, PP, and PEP are not concomitant measures; for this reason and simple use, arm cuff blood pressure measurement was not reduced by 0.9 as to approximate the LV pressure.
—VAC/LVEF: considering that the ideal theoretical value of VAC/LVEF is ≈1, this ratio intends to measure how far a particular situation deviates from the ideal value.

For the statistical analysis, we used the online statistic tool site www.statskingdom.com and SPSS Inc statistical software, version 2020. Continuous variables were expressed as mean +/- SD and categorical variables as percentages. Variables were tested for normal distribution. Patient characteristics were compared using Fisher's exact test for categorical variables and the independent *t*-test for normally distributed continuous variables. Correlations were made by the Pearson method.

Among the new indices, we searched for the most discriminant between the A and B groups as well the one with the best correlation with Ees and or VAC. Simple and multiple correlations with the Chen's Ees formula were investigated, with the aim to understand how close to the formula we can get, based on simple measurements and simple operations. Regression line equations were computed only for high R values of at least 0.9. For the regression models we used the following metrics:

a. For the degree of determination were calculated: R Square (*R*^2^) and for multiple regression model the adjusted *R*^2^ (*R*^2^ adj.) as to measure the degree of variability in the dependent variable, explained by the model and, respectively eliminate by the *R*^2^ adj. an eventual overfitting problem brought by an independent variable.
b. Right-tailed goodness of fit, Mean Square Error (MSE), and the Root Mean Square Error (RMSE) were performed.
c. Multicollinearity was eliminated by a variance inflation factor < 2.5.
d. Natural logarithm (Ln) regression and the derived power regression were calculated as a companion of the original formulas.
e. A weighted regression was preferred if an inhomogeneity of variance was detected, expressing “*y*” as its natural logarithm Ln(*y*).

## Results

Table 1 depicts the demographical and clinical characteristics of the studied groups. No significant difference was observed between the groups, for age, body surface area, or heart rate.

**Table 1:** Demographical and clinical characteristics of the studied groups.

	**Group A** ***n =*** **12**		**Group B** ***n =*** **21**		***p***
	**Mean** **values**	**Std** **dev**	**Mean** **values**	**Std** **dev**	
**Age (y)**	62.58	11.67	63.14	9.97	NS
**BSA (m** ^**2**^ **)**	1.89	0.18	1.93	0.16	NS
**Heart Rate (b/min)**	64.83	6.79	69.9	13.27	NS
**Hemoglobin level (g/dl)**	13.4	0.6	12.7	1.4	NS
**SBP (mmHg)**	135	17	117	20	0.0135
**DBP (mmHg)**	77	12	71	14	0.2227
**Pulse Pressure (mm Hg)**	58.5	11.84	45.71	13.52	<0.001
**Ischemic etiology (%)**	41		57		NS
**RASI (%)**	50		85		0.0441
**BB (%)**	41		85		0.0164

*BSA, body surface area; RASI, 50% of guideline renin-angiotensin system inhibitor recommended dose; BB, 50% of guideline beta-blocker recommended dose; SBP, Systolic Blood Pressure; DBP, diastolic blood pressure*.

**Figure 1:**
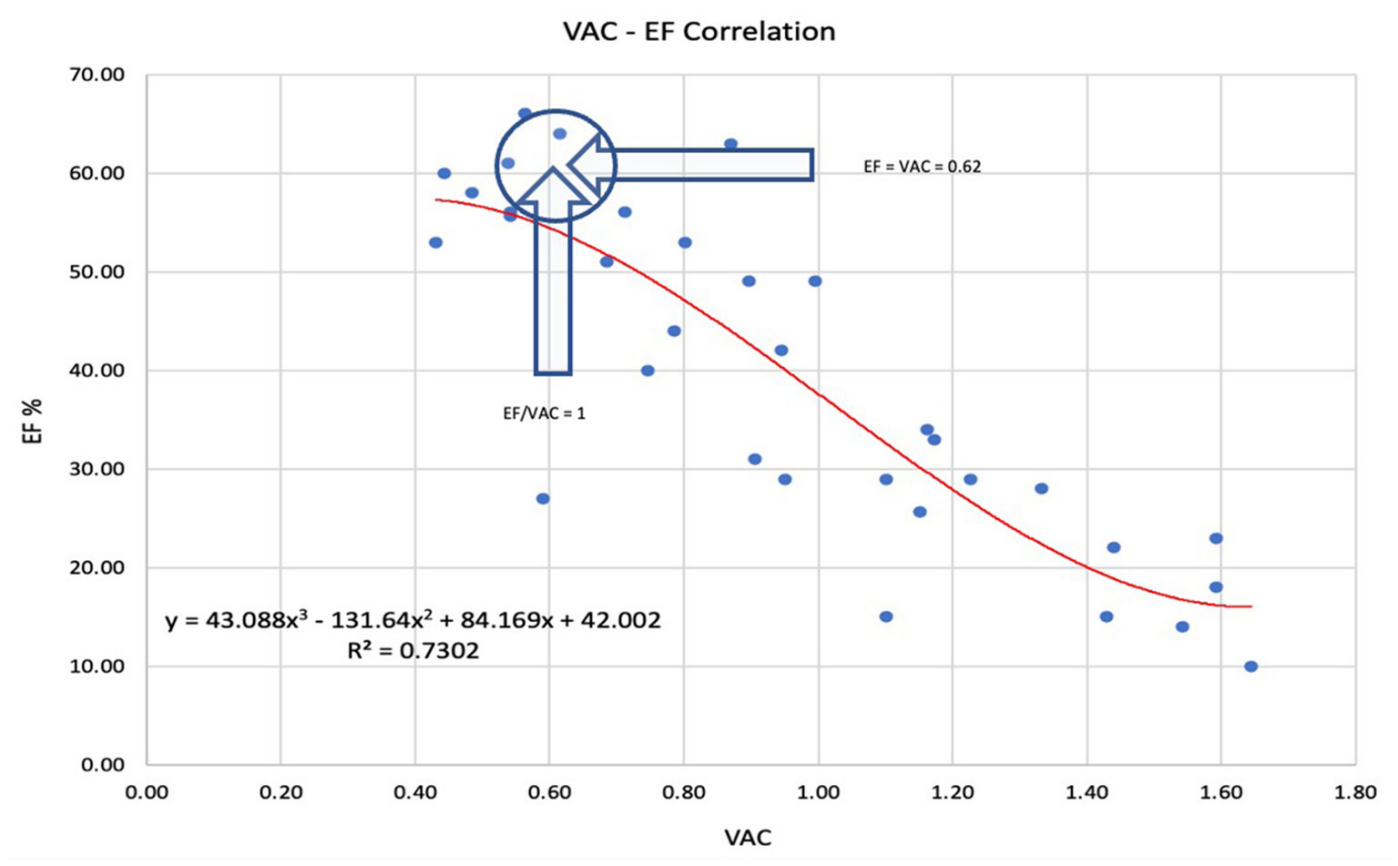
The best prediction of the left ventricular ejection fraction (LVEF) to ventricular arterial coupling (VAC) relationship was obtained by 3rd-degree polynomial regression. The arrows indicate the point where LVEF = VAC = 0.62 and VAC/LVEF = 1. LVEF Left ventricle ejection fraction, VAC ventriculo-arterial coupling.

**The LVEF to VAC relationship**: A strong negative nonlinear correlation was observed. The best *R*^2^ (0.73) was obtained with a 3rd-degree polynomial relationship (Figure 1). The intersection of the tendency curves was at a value of 0.61, where LVEF and VAC are equal, regardless of the curve type (linear, logarithmic, or polynomial).

**Figure 2:**
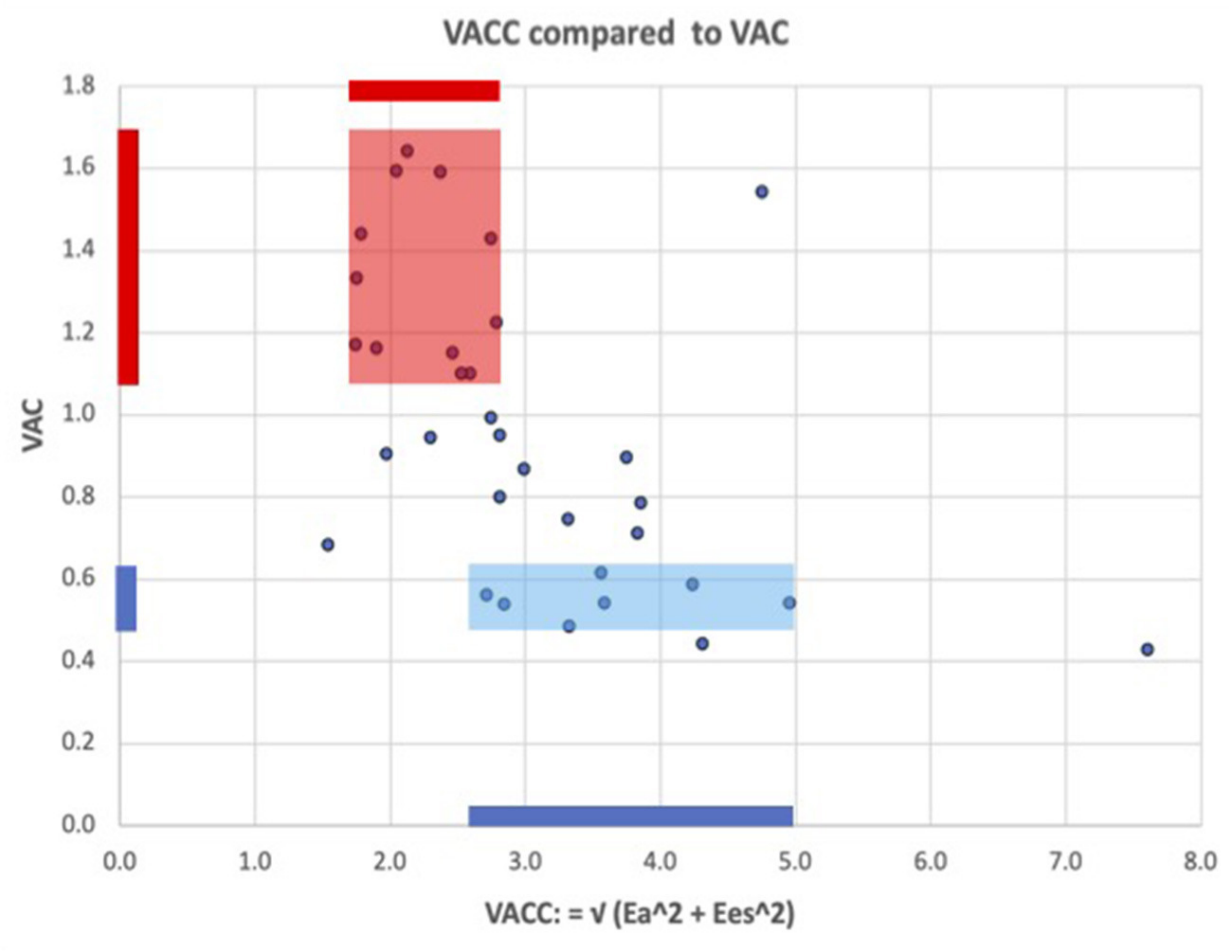
ventricular arterial coupling and its companion: VACC = √(Ea^2^ + Ees^2^). The fairly low correlation shows that VAC and VACC partly reflect independent information, i.e., VAC alone cannot adequately describe the arterio-ventricular coupling system. Blue color: for patients with a normal VAC, VACC has wide variations and greater values than in patients with abnormal elevated VAC (red color), where VACC has lesser values and more limited variations.

The VAC correlation to its companion VACC = √(Ea^2^ + Ees^2^) demonstrated a significant negative weak correlation (*R* = −0.51; *R*^2^ = 0.26; *R*^2^_(ln)_ = 0.28; *P* = 0.002). The weak correlation indicates that VACC may be considered an independent predictor. Figure 2 shows greater values and a wide variation of the VACC for patients with normal VAC, while in patients with abnormal VAC elevation, the VACC has lesser values and limited variation.

**Figure 3:**
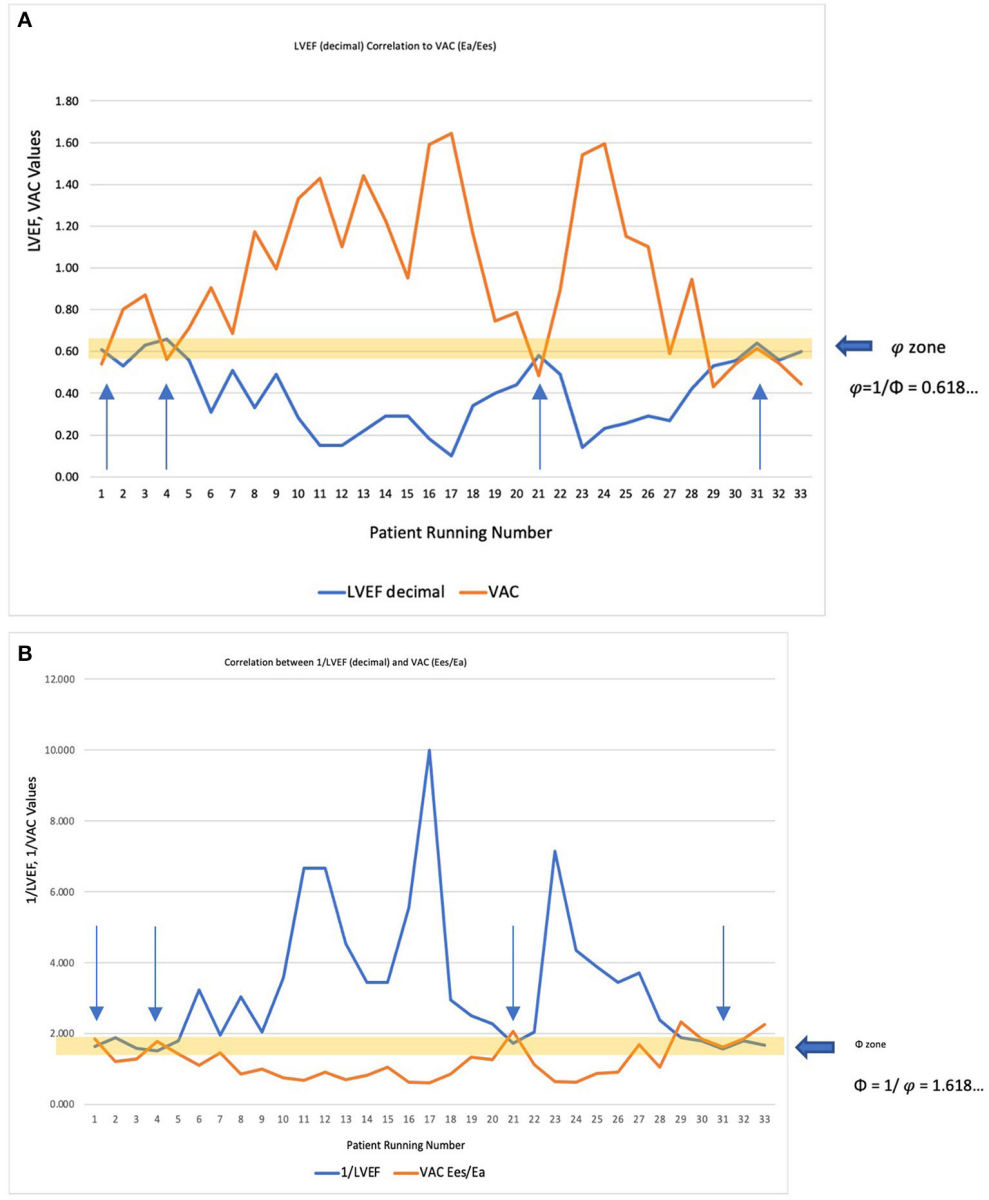
**(A)** LVEF decimal (<1) correlated to the most used VAC formula (Ea/Ees). The “φ zone” is the narrow interval very close to the optimal value (φ≈0.618…), where LVEF = VAC = 0.62. The arrows indicate the four patients within this interval. **(B)** Considering that ventricular elastance (Ees)/arterial elastance (Ea) is the reciprocal ratio of the Ea/Ees, the correlation was made with 1/LVEF (decimal). The “Φ zone” will be therefore in the close vicinity of 1.618 (Φ), where 1/LVEF = Ees/Ea ≈ 1.618. The arrows indicate the same four patients within this interval.

Commonly expressed by the ratio Ea/Ees, some authors define the VAC by its reciprocal ratio: Ees/Ea, meaning $\frac{1}{\frac{Ea}{Ees}}$. Comparative correlations are displayed in Figures 3A,B. If for the most used VAC ratio (Ea/Ees) the ideal situation is when VAC = LVEF = 0.62 (approximating φ≈0.618…), its reciprocal ratio (VAC = Ees/Ea) becomes 1/0.62 ≈1/φ≈1/0.618… ≈1.618…(Φ). The “φ or Φ zone”, close to the optimal values, is displayed on both Figures 3A,B), showing the same 4 patients inside the optimal zone.

Both ratios can be correlated to the same variables obtaining different coefficients. However, all the following results are based on the most commonly used VAC formula: Ea/Ees.


$$\begin{array}{l} The\;VAC\;formula\;can\;be\;simplified:\\ \frac{Ea}{Ees}=\frac{\frac{ESP\left(mmHg\right)}{SV\left(ml\right)}}{\frac{ESP\left(mmHg\right)}{ESV-V0\left(ml\right)}}=\frac{ESV-V0\left(ml\right)}{SV\left(ml\right)}. \end{array}$$

**Figure 4:**
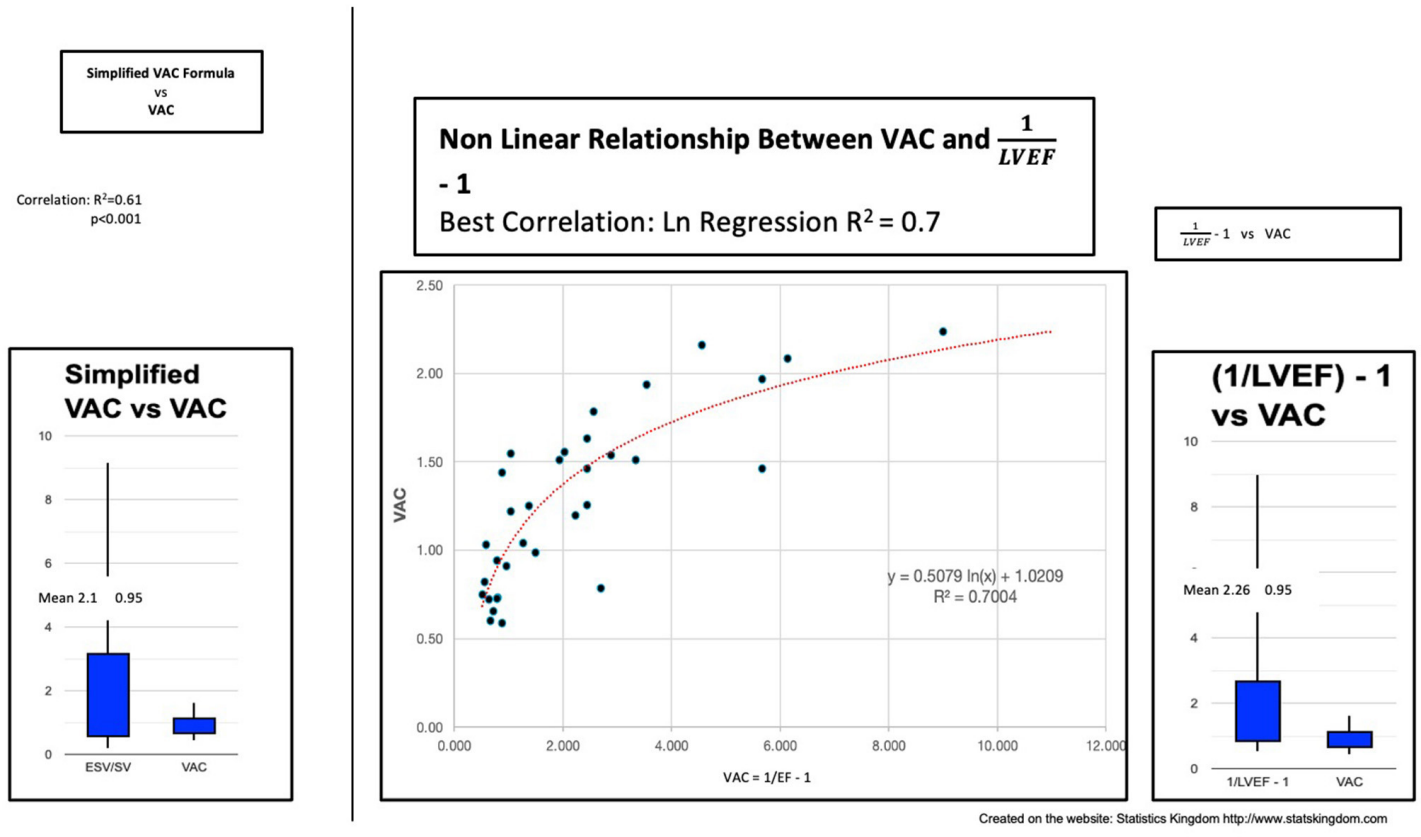
**Left**: Simplified VAC (ESV/SV) vs. VAC; **Center:** Curvilinear (logarithmic) regression between (1/LVEF) – 1 and VAC; Right: VAC calculated by (1/LVEF) – 1 vs. VAC ESV left ventricle end-systolic volume, SV stroke volume, VAC ventricular-arterial coupling, LVEF left ventricular ejection fraction.

If we consider V_0_ = 0 ml, we obtain $\frac{ESV\left(ml\right)}{SV\left(ml\right)}$. In our study, this dimensionless ratio heavily overestimated the VAC, despite having a significant correlation—the *R*^2^ value was only 0.61, meaning a good prediction of only 61% (Figure 4).

Can the VAC be predicted by “(1/LVEF) – 1”? In our study, we found the same important overestimation of the VAC and the weak power of prediction: *R*^2^ (linear) 0.63 or *R*^2^_(ln)_ 0.7 demonstrating a better fit with a nonlinear function (Figure 4).

### Indices of Cardiovascular Hemodynamics Calculated by Transthoracic Echocardiography

**Figure 5:**
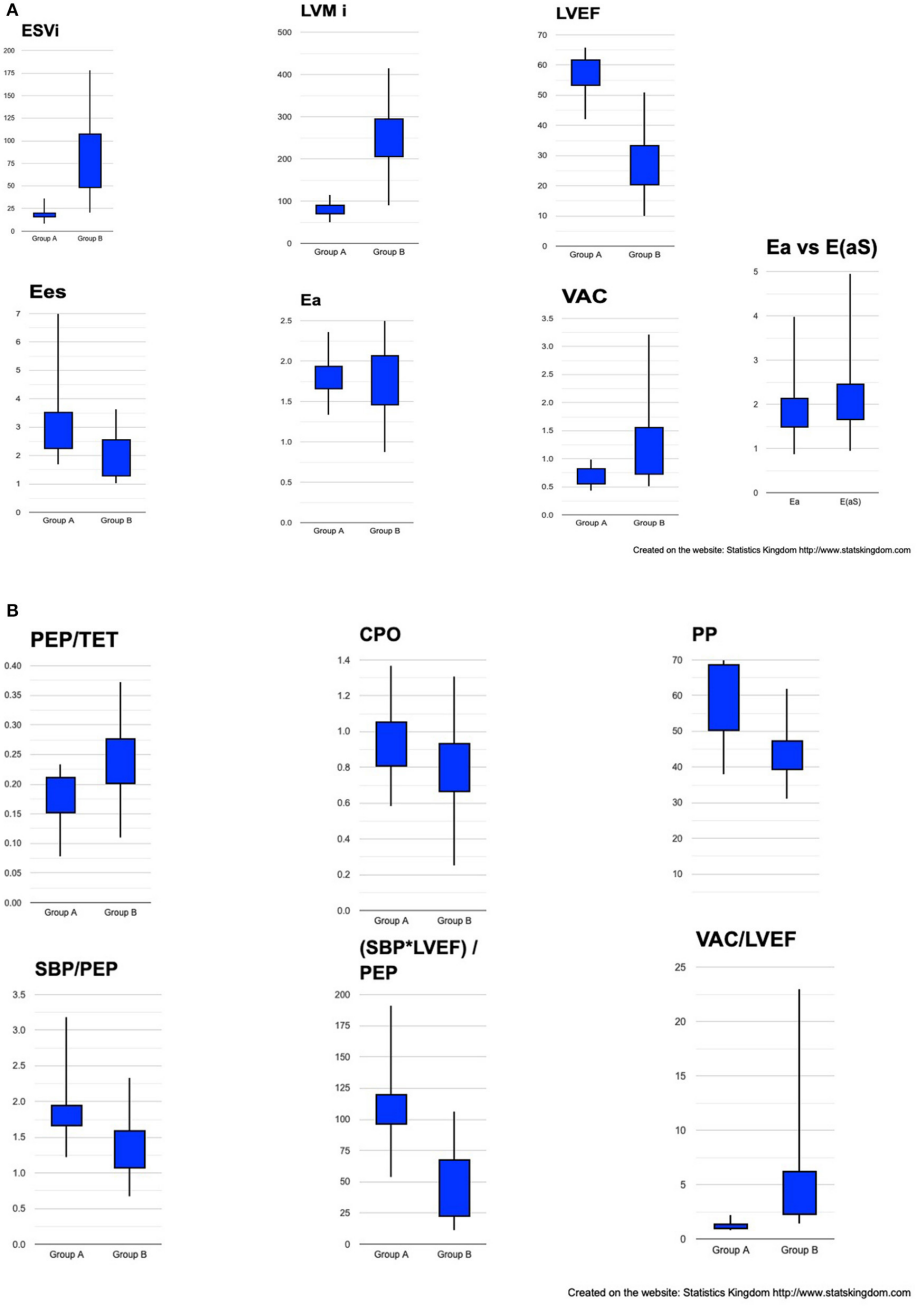
Box plot representation of cardiac structural and hemodynamic parameters among groups. **(A)** ESVi, LVMi, LVEF, Ees, Ea, VAC, Ea vs. E(aS). **(B)** PEP/TET, CPO, PP, SBP/PEP, (SBP*LVEF)/PEP, VAC/LVEF. ESVi, indexed LV end-systolic volume; LVMi, indexed LV mass; LVEF, LV ejection fraction; Ees, ventricular elastance; Ea, effective arterial elastance; VAC, ventricular-arterial coupling; E(aS), arterial elastance calculated by Segers formula; PEP, pre-ejection period; TET, Total ejection time; CPO, cardiac power output; PP, pulse pressure; SBP, systolic blood pressure.

The two groups were significantly separated by the structural and hemodynamic parameters listed in Table 2. As expected, the ESVi and indexed LVM were significantly greater in group B. LVEF, VAC, VAC/LVEF, and CPO did make a significant separation but CPO only at a minimal mean value difference. The VAC/LVEF ratio measures how far from the theoretical normal value will a particular situation deviate, considering that the theoretical perfect VAC to LVEF ratio is very close to 1, where V0 = 0 ml (23). Mean values for Ees were significantly lower in group B but with important overlapping margins; Ea had equal means. All the systolic time intervals showed differences, the PEP being at the limit of signification. Figures 5A,B depicts the boxplot chart for the relevant indices. The ratios based on pressure, time and LVEF, also made clear high significant separations among the groups. From the easiest to measure to the more complex indices, the best discriminators were: ESVi, (SBP^*^LVEF)/PEP, and VAC/LVEF.

**Table 2:** Hemodynamic variables in the two groups.

**Total group** ***N =* 33**	**Group A** ***n =*** **12**		**Group B** ***n =*** **21**		***p***
	**Mean values**	**Std dev**	**Mean values**	**Std dev**	
**ESVi (ml/m** ^**2**^ **)**	18.54	7.91	80.68	43.16	<0.001
**LVMi (mass (g/m** ^**2**^ **)**	75.85	31.32	114.96	53.52	0.005
**LVEF (%)**	56.55	6.83	29.75	12.85	<0.001
**Ea (mm Hg/ml)**	1.9	0.46	1.9	0.63	NS
**Ees (mm Hg/ml)**	3.13	1.45	1.84	0.79	0.012
**VAC**	0.67	0.19	1.20	0.67	0.002
**PEP (ms)**	70.08	23.22	89.67	27.96	0.039
**LVET (ms)**	327.17	27.62	271.19	44.83	<0.001
**PEP/TET**	0.176	0.046	0.243	0.069	0.002
**CPO (W)**	0.92	0.21	0.79	0.25	0.007
**SBP/PEP (mm Hg/ms)**	2.27	1.33	1.47	0.66	<0.001
**PP/PEP (mm Hg/ms)**	0.97	0.57	0.58	0.3	<0.001
(SBP*LVEF)/PEP (mm Hg/ms)	128.35	71.96	45.84	29.92	0.002
**VAC/LVEF**	1.22	0.49	5.5	5.31	0.001
**Ea: (SBP/SV) vs. Segers Arterial Elastance formula:** **(−0.127 + 1.02R/T + 0.314/C)**	Ea: SBP/SV		Ea(Segers): (−0.127 + 1.02R/T + 0.314/C)		
	Mean value: 1.9	Std. dev.: 0.56	Mean value: 2.13	Std. dev.: 0.71	NS *p =* 0.14923458

*ESVi, indexed LV end-systolic volume; LVMi, indexed LV mass; LVEF, left ventricular ejection fraction; Ea, effective arterial elastance; Ees, ventricular elastance; VAC, ventricular-arterial coupling; PEP, pre-ejection period; LVET, LV ejection time; TET, total ejection time; CPO, cardiac power output; SBP, Systolic Blood Pressure; PP, Pulse Pressure; SV, stroke volume; R, arterial resistance; T, cardiac cycle duration; C, compliance; NS, not-significant*.

Comparison of the simplified Ea formula to Segers arterial elastance formula E_(aS)_. No significant difference was found between the two formulas (Table 2; Figure 5A). The correlation between Ea and E_(aS)_ was almost perfect (*R* = 0.985; *R*^2^ = 0.97; *P* = 0).

**Figure 6:**
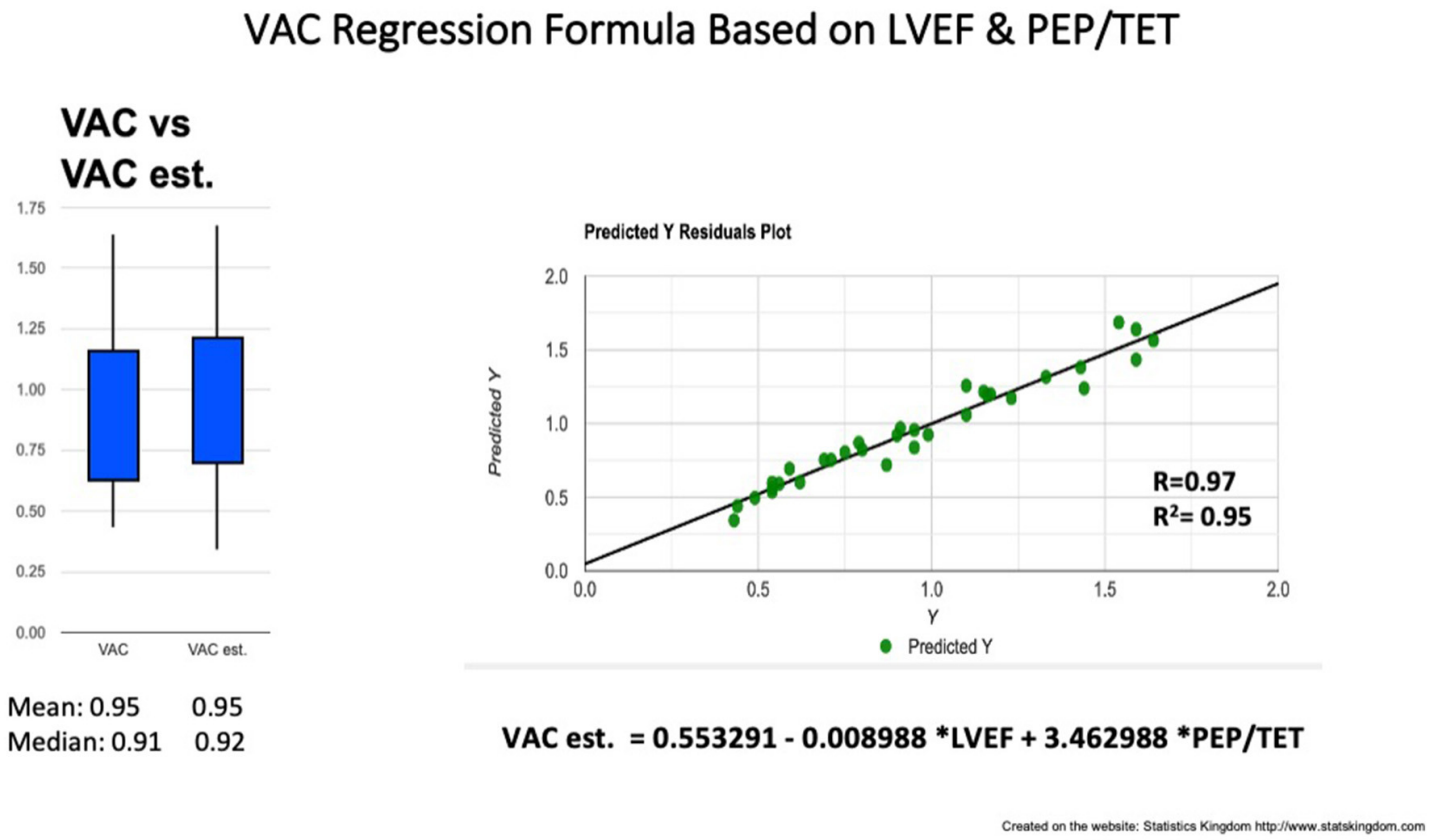
VAC prediction by multiple regression formula based on LVEF and PEP/TET. Left: Box plot comparison between VAC and estimated (est.) VAC; Right: Correlation line VAC to VAC est. VAC ventriculo-arterial coupling, LVEF left ventricle ejection fraction, PEP pre-ejection period, TET total ejection fraction.

Correlations between simple measurements and the VAC (calculated on the basis of Ea and Chen's Ees formula). Analyzing the overall group, the VAC was best correlated to PEP/TET (*R* = 0.93; *R*^2^ = 0.86) and the LVEF (*R* = −0.85, *R*^2^ = 0.72) (Table 3). The simple regression equation: −0.128 + 4.957 ^*^ PEP/TET, predicts 86% of the variables. Combining PEP/TET and LVEF, by multiple regression, the VAC was strongly predicted by the formula: *VAC est*. = *0.553291 – 0.008988* ^*^ *LVEF* + *3.462988* ^*^ *PEP/TET* (*R* = 0.97; *R*^2^ = 0.95) (Table 4; Figure 6), leading to a dimensionless result. Comparison between the VAC and the VAC est. (Figure 4: Box plot) did not find significant differences having the same mean values.

**Table 3:** Correlation analysis in the overall group between main hemodynamic indices.

**Correlations**	**Total group (33 pts)**			
	***R***	**R^**2**^**	***p***	**MSE/RMSE**
LVEF to VAC	−0.8499	0.7223	<0.001	0.0371/0.1926
PEP to VAC	0.8127	0.6604	<0.001	–
VACC to VAC	−0.51	0.26	0.002	–
PEP/TET to VAC	0.9258	0.8571	<0.001	0.019/0.1379
VAC = −0.128+4.957*PEP/*TET*				
(SBP*LVEF)/PEP to VAC	−0.7912	0.6261	<0.001	–
LVEF to Ees	0.5223	0.2728	0.001	–
ESVi to Ees	−0.5157	0.266	0.002	–
PEP to Ees	−0.7232	0.523	<0.001	–
PEP/TET to Ees	−0.7229	0.5226	<0.001	–
(SBP*LVEF)/PEP to Ees	0.9213	0.8488	<0.001	0.2209/0.47
$Ees=0.9482+0.01796*\frac{SBP*LVEF}{PEP}$				
SBP/PEP to Ees	0.9159	0.8389	<0.001	0.2353/0.4851
$Ees=0.364796+1.105611*\frac{SBP}{PEP}$				

*LVEF, left ventricular ejection fraction; VAC, ventricular-arterial coupling; PEP, pre-ejection period; VACC, VAC companion = √(Ea^2^+Ees^2^); Ees, ventricular elastance; PP, pulse pressure; SBP, arm cuff systolic blood pressure; TET, total ejection time; SV, stroke volume; ESVi, indexed left ventricular end-systolic volume; MSE, Mean Squared Error; RMSE, Root Mean Squared Error*.

**Table 4:** Multiple regressions for Ees and VAC prediction.

**Multiple regressions: Total group (33 pts)**				
***X***, ***Y***	***R***	**R** ^**2**^	***p***	***Y*** **Variance**
**Multiple regression: Ees predicted by DBP, SV, PEP and TET**				
X1: DBP X2: SV X3: PEP X4: TET Y: Ees	0.952	0.907 *R*^2^ adj. 0.893	<0.001	0.221917
	$Ees\left(mmHg/ml\right)=0.00164628*\frac{DBP^{1.017059}*TET^{1.904122}}{SV^{0.807318}*PEP^{1.193503}}$ (mmHg/ml)			
**Multiple regression: VAC predicted by LVEF and PEP/TET**				
**X1: LVEF** **X2: PEP/TET** **Y: VAC**	**0.975182**	**0.950980** *R*^2^ adj. **0.947712**	<0.001	0.13678
	Multiple Regression Equation (unitless):			
	VAC = 0.553291−0.008988*LVEF+3.462988*PEP/*TET*			
	Ln/Power Regression Equation:			
	*VAC* = 9.309923·*LVEF*^−0.321084^·*PEP*/*TET*^0.767997^(*R*^2^ = 0.93;*R*^2^*adj*. = 0.92)			
**Ees derived (Ees_d) from the VAC formula**:				
(If VAC = Ea/Ees, then Ees = Ea/VAC)				
$\to Ees\text{\_}d\approx \;\frac{Ea}{0.553291-0.008988*LVEF+3.462988*PEP/TET}\;=\;\frac{SBP}{SV*\left(0.553291-0.008988*LVEF+3.462988*\frac{PEP}{TET}\right)}$(mmHg/ml)				
	0.9734	0.9474	<0.001	1.5064

*Ln, natural logarithm; DBP, diastolic blood pressure; SV, Stroke volume; PEP, pre-ejection period; TET total ejection time; Ees, ventricular elastance; VAC, ventricular-arterial coupling; LVEF, left ventricular ejection fraction*.

In search of a third VAC determinant, we added the DBP/SBP ratio to LVEF and PEP/TET. As to avoid a homoscedasticity statistical bias, a weighted regression was needed, leading to the following result: Ln (VAC) = 0.147279 – 1.456324^*^DBP/SBP^*^0.9 – 0.0103058^*^LVEF + 4.206581^*^PEP/TET (*R* = 0.987; *R*^2^ = 0.975; *P* = 0).

### Correlations Between Simple Measurements and Ees Chen's Formula

Left ventricular ejection fraction was a significant (*P* = 0.002) but poor determinant for Ees (*R*^2^ = 0.27) meaning that only 27% of the Ees formula is dependent on the LVEF. On the other hand, the simple SBP/PEP ratio was significantly correlated to Ees (*P* < 0.001, R = 0.92, *R*^2^ = 0.84). The resulting regression equation of the Ees derived (Ees_d): Ees_d (mm Hg/ml) = 0.364796 + 1.105611^*^ SBP/PEP (mm Hg/ml).

As LVEF correctly predicts HF severity, we integrated LVEF (as a dimensionless ratio) into the SBP/PEP formula in order to combine the predictability of the LVEF to the determinant role of SBP/PEP on the Ees variations, obtaining: $\frac{SBP^{*}LVEF}{PEP}$ mmHg/ms. The *R*^2^ value (*R*^2^ = 0.85) was not significantly improved. However, the result is a hybrid index, cumulating the positive/negative predictive value for the severity of disease but also being a strong predictor of the Ees value. For example, if we take a 70 mm Hg/ms cutoff, the positive predictive value in our group was 89% and the negative predictive value 71%. At the same time, this ratio keeps a very strong determinant correlation to the Ees formula but also a good significant correlation to VAC (*R* = −0.79; *R*^2^ = 0.62) possibly representing a good and simple index of cardiac function.

### Finding the Easiest Prediction for Chen's Ees Formula

Considering the VAC formula: VAC = Ea/Ees, then Ees = Ea/VAC.

If we replace the VAC with the VAC est., we obtain Ees_d ≈ Ea/VAC est. The VAC est. predicted the VAC with a risk of error of 5% (*R*^2^ = 0.95). Replacing the VAC by the VAC est. should lead to the same risk of error for the Ees prediction.

Then, Ees_d (mm Hg/ml) ≈


$$\begin{array}{ll} \approx & \frac{Ea\;=\;\left(\frac{SBP\;*\;0.9}{SV}\right)}{VACest.\;=\;\left(0.553291-0.008988\;*\;LVEF+3.462988\;*\;\frac{PEP}{TET}\right)}\\ = & \;\frac{SBP\;*\;0.9}{SV^{*}\;\left(0.553291-0.008988\;*\;LVEF+3.462988\;*\;\frac{PEP}{TET}\right)} \end{array}$$

**Figure 7:**
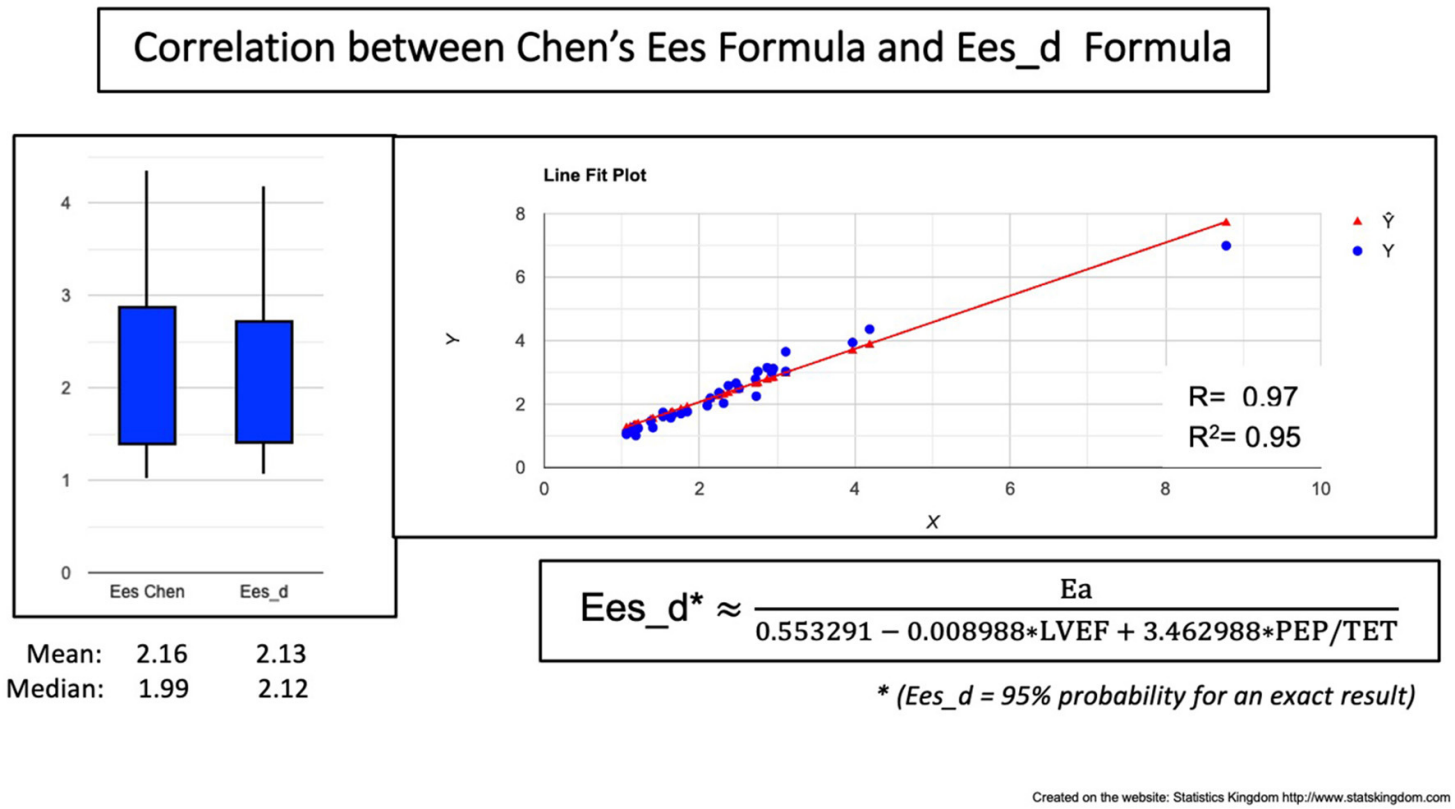
Correlation between Chen's formula and the VAC-Derived Ees formula (Ees_d). Left: Boxplot demonstrating the similar values; Right: Correlation line.

Ees_d correlated, to Chen's Ees formula with *R* = 0.97 and *R*^2^ = 0.95 meaning a 5% risk of error (Figure 7). No significant differences were observed between Chen's Ees and Ees_d obtained values (Figure 7, box plot).

## Discussion

As HF is mainly a clinical diagnosis and its prognosis and burden of disease are mainly determined by biological and clinical characteristics (44), we believe that the understanding of hemodynamic parameters should not be uncoupled from symptoms. The staging classification of HF recognizes HF as a continuum in relation to the occurrence of symptoms (1, 45). On the other hand, the common clinical practice focuses on characterizing symptomatic HF by limited indices such as LVEF, filling pressures, cardiac output for main diagnostic and therapeutic purposes (46). A rather homogeneous group of patients with severe HF from the point of view of symptoms and burden of clinical disease (as was our group B) can be inhomogeneous in regard to the degree of cardiac function alteration, hence the necessity of accounting for the dynamic relation between heart and peripheral response (such as Ees/Ea for VAC calculation or systolic time intervals and blood pressure). Previously published study on invasively determined PVL, conclusively showed that patients in stages A and B share similar hemodynamic profiles, with overlapping PVL; stages C and D patients were also hemodynamically similar, although more heterogeneous; Ees was the most important parameter to significantly different clinical profiles (47).

We focused our analysis on patients with severe HF with reduced EF (group B). As these patients had dilated, remodeled LV, less adapted to the arterial load, the highly significant differences when compared to asymptomatic patients, are not surprising. A good hemodynamic parameter, relevant for clinical practice should perform well in separating such patients, and should also give a measure of the dynamics of severity. Despite its limitations, LVEF remained a powerful discriminator between groups A and B. This result represents a common clinical expectation since group B contains heavily diseased hearts. Simple ratios proved their usefulness: SBP/PEP was a good discriminator between the two groups and a very good predictor for the Ees. As SBP tends to be lower in patients with HFrEF and PEP to be longer, their ratio will be a natural good discriminator. Taking advantage of the dimensionless LVEF ratio, we could integrate LVEF into the ratio (LVEF^*^SBP/PEP) (mm Hg/ms) without modification of the units. In patients with HFrEF, SBP tends to be lower and PEP longer. Multiplying the SBP/PEP by an expected lower than normal LVEF will further accentuate the difference from normal. The obtained ratio (SBP^*^LVEF)/PEP (mm Hg/ms) maintained the same good predictability for the Ees (*R*^2^ = 0.85) and at the same time was a powerful discriminator between group A and group B. With these two benefits, this ratio could be a simply available way for patients' evaluation.

The VAC/LVEF ratio (meaning Ea/(Ees^*^LVEF) ratio), which is harder to estimate, measures the degree of deviation from the normal value which is around 1. This ratio was the most discriminant, with a mean value 4.5 times greater in group B compared to A. While the LVEF and VAC might be inside the normal ranges, the ratio between them might point out a relative anomaly. Could it be useful for borderline cardiac dysfunction? Further studies are needed for further validation, long-term follow-up, and therapeutic response.

Ea did not demonstrate any difference. Considering Group B stronger medication (RASI, BB) this equality might have at least a partial explanation. Comparing the mean values, Ees was moderately lower in group B (−41%)—at the limit of statistical difference (*P*: 0.012) not being a trustworthy discriminant factor. The VAC had a very significant increment of 79% in group B. Is the LVEF a strong or weak determinant for the VAC? In the total group, LVEF and VAC had a significant negative correlation (*P* < 0.001) but R-Square (*R*^2^) equals only 0.74, which means that only 74% of the variability of the VAC can be explained by the LVEF—and this correlation was obtained by a nonlinear, 3rd-degree polynomial equation (Figure 1). At the same time, the tendency curves demonstrated that: a. the LVEF and VAC will intersect at 0.6, supporting the mathematical issue by which LVEF and VAC are equal at the value of 1/Phi (0.618…) and b. that for a wide range, LVEF—VAC relationship cannot be expressed by a simple linear equation (23). The VACC had a negative significant correlation to the VAC but the low *R*^2^ suggests that VACC could be an independent predictor, as displayed in Figure 2.

In search of simple, ready-to-use clinical indices and an easier approach, this study made correlations and regression equations relative to Chen Ees formula and the derived VAC (using, for this reason, the same variables) but not to invasively calculated LV elastance. Ees single-beat formulas are based on systolic times, blood pressure, and volumes. The PEP and TET play a central role in Ees calculation and the derived VAC. In our group of patients, the arterial elastance surrogate (Ea = SBP/SV) had minimal non-significant differences, compared to a more complex arterial elastance formula (39) with one limitation: if the extreme heart rates (HR) were between 54 and 98/min, the vast majority were between 61 and 73/min—meaning that we had a low HR variation leading to a lower variation of the arterial impedance. As our study addresses patients under medication with a controlled HR, we conclude so far, that the simple surrogate Ea seems a reliable index.

The two variables with the best correlation to VAC were PEP/TET and LVEF. Combining them, led to this multiple regression equation = *0.553291 – 0.008988 LVEF* + *3.462988 PEP/TET*, giving very strong predictability of 95%. By adding DBP/SBP to the multiple regression, we obtained an almost perfect correlation (*R* = 0.987; *R*^2^ = 0.975) to Ln(VAC) demonstrating the determinant role of the three dimensionless ratios (LVEF, DBP/SBP, and PEP/TET). However, this very demonstrative formula is limited to the VAC natural logarithm and not to a decimal number. Maybe on a larger group of patients, it could be expressed in a more practical manner.

Ees formula had a weak positive correlation with LVEF and a negative one with ESVi. Being better correlated to PEP and PEP/TET and PP, it invites to combine pressure, time, and volume to create a composite index containing the roots of Chen's formula. The resulting correlations were very strong, covering a group of patients with very high variations of cardiac function. Adding the common measures DBP, SV, PEP, and TET, led to a very strong correlation.

A question must be raised: if the VAC estimation is very close to the VAC calculated through Chen's Ees formula, do we need a further deduction of the Ees? However, a very simple way was found: by replacing the original VAC with the formula: “*VAC* = *0.553291 – 0.008988 LVEF* + *3.462988 PEP/TET*”, we took a 5% risk of error for the Ees prediction. This was true since the obtained Ees_d predicted Chen's formula at *R*^2^ = 0.95. This study highlights that systolic time intervals are a forgotten gold mine. The ratio pressure over time, as the dp/dt ratio, is a major index of myocardial contractility. Our measurements are not concomitant, being therefore close but not equal to dp/dt; however the SBP/PEP ratio had very strong correlations to Ees. Otherwise, single-beat surrogates for Ees calculation were developed based on systolic times, pressure, and volumes. Shishido's single-beat formula seems to be superior to Chen's formula but needs LV end-diastolic pressure estimation (48) adding, therefore, for non-invasive studies, an approximation. Our linear correlations to a linear formula surrogate of the Ees, cannot cover the complexity of the Ees. Considering that Chen's formula may have significant variations for minimal PEP measurement differences, our approach constructed on basic measurements without exponential developments, integrated into easy-to-use formulas, might represent at least a valuable preliminary/complementary information on the LV contractility.

## Limitations

The patient dataset is not representative of the global HF population. HF with preserved EF (HFpEF) was not represented, as we focused on HF with reduced EF. This study did not analyze normal subjects; however, the group of patients is representative of a cardiology department. Against the small number of patients, we were committed to elaborate statistical work. Relative to the Ees and VAC, this study is limited to an easier approach of the Chen formula, which despite invasive validations, remains an approximation of the LV elastance. The new formulas, validated by our study, still need further validation in the future. Our study might be considered an incentive for further studies on greater groups of patients, including documented HFpEF patients.

## Conclusion

Heart failure (HF) hemodynamics is only basically characterized in clinical everyday practice. Dimensionless ratios, such as LVEF, have certain advantages which explain widespread use, while the VAC is underused being limited by the Ees calculation difficulty. Basic measurements were found as valuable determinants for the VAC. We suggest overcoming their disadvantages by incorporating other meaningful parameters such as blood pressure, systolic time intervals, and SV that are rough, unprocessed physiological data, easy to understand, and measure. We have shown that the simple ratio between SBP and PEP can be very informative in patients with HF being very strongly correlated to Chen's Ees formula. At the same time, Chen's formula and the derived VAC can be well predicted by simple-to-use measurements, closing the gap between too complex formulas and the daily practice.

## Data Availability Statement

The raw data supporting the conclusions of this article will be made available by the authors, without undue reservation.

## Ethics Statement

The studies involving human participants were reviewed and approved by Emergency Institute for Cardiovascular Diseases, Bucharest. The patients/participants provided their written informed consent to participate in this study.

## Author Contributions

E-LA and SM equally contributed in all aspects of conception, design, statistical analysis, interpretation, and writing of the manuscript. OC contributed to the writing and revision of the manuscript. All authors contributed to manuscript revision, read, and approved the submitted version.

## Conflict of Interest

The authors declare that the research was conducted in the absence of any commercial or financial relationships that could be construed as a potential conflict of interest.

## Publisher's Note

All claims expressed in this article are solely those of the authors and do not necessarily represent those of their affiliated organizations, or those of the publisher, the editors and the reviewers. Any product that may be evaluated in this article, or claim that may be made by its manufacturer, is not guaranteed or endorsed by the publisher.
